# Clinically significant prostate cancer (csPCa) detection with various prostate sampling schemes based on different csPCa definitions

**DOI:** 10.1186/s12894-021-00949-7

**Published:** 2021-12-23

**Authors:** Fei Wang, Tong Chen, Meng Wang, Hanbing Chen, Caishan Wang, Peiqing Liu, Songtao Liu, Jing Luo, Qi Ma, Lijun Xu

**Affiliations:** 1 Department of Ultrasound, The Second Affiliated Hospital of Soochow University, 1055 Sanxiang Road, Suzhou, Jiangsu China; 2 Department of Urology, The Second Affiliated Hospital of Soochow University, 1055 Sanxiang Road, Suzhou, Jiangsu China

**Keywords:** Clinically significant prostate cancer, Contralateral, Ipsilateral, Systematic biopsy, Targeted biopsy

## Abstract

**Background:**

Combining targeted biopsy (TB) with systematic biopsy (SB) is currently recommended as the first-line biopsy method by the European Association of Urology (EAU) guidelines in patients diagnosed with prostate cancer (PCa) with an abnormal magnetic resonance imaging (MRI). The combined SB and TB indeed detected an additional number of patients with clinically significant prostate cancer (csPCa); however, it did so at the expense of a concomitant increase in biopsy cores. Our study aimed to evaluate if ipsilateral SB (ipsi-SB) + TB or contralateral SB (contra-SB) + TB could achieve almost equal csPCa detection rates as SB + TB using fewer cores based on a different csPCa definition.

**Methods:**

Patients with at least one positive prostate lesion were prospectively diagnosed by MRI. The combination of TB and SB was conducted in all patients. We compared the csPCa detection rates of the following four hypothetical biopsy sampling schemes with those of SB + TB: SB, TB, ipsi-SB + TB, and contra-SB + TB.

**Results:**

The study enrolled 279 men. The median core of SB, TB, ipsi-SB + TB, and contra-SB + TB was 10, 2, 7 and 7, respectively (P < 0.001). ipsi-SB + TB detected significantly more patients with csPCa than contra-SB + TB based on the EAU guidelines (P = 0.042). They were almost equal on the basis of the Epstein criteria (P = 1.000). Compared with SB + TB, each remaining method detected significantly fewer patients with csPCa regardless of the definition (P < 0.001) except ipsi-SB + TB on the grounds of D1 (P = 0.066). Ten additional subjects were identified with a higher Gleason score (GS) on contra-SB + TB, and only one was considered as significantly upgraded (GS = 6 on ipsi-SB + TB to a GS of 8 on contra-SB + TB).

**Conclusions:**

Ipsi-SB + TB could acquire an almost equivalent csPCa detection value to SB + TB using significantly fewer cores when csPCa was defined according to the EAU guidelines. Given that there was only one significantly upgrading patient on contra-SB, our results suggested that contra-SB could be avoided.

**Supplementary Information:**

The online version contains supplementary material available at 10.1186/s12894-021-00949-7.

## Background

The number of patients with prostate cancer (PCa) has increased in the last decades in the USA. An estimated 174,650 and 191,930 men were diagnosed with PCa in 2019 and 2020, respectively, and the number of related deaths was 31,620 and 33,330, respectively [1, 2]. PCa is the only tumor diagnosed by blindly puncturing the entire organ rather than just the identified lesion by imaging due to the considerable overlap between benign and malignant lesion appearances in the imaging [3]. Despite its relatively low sensitivity (39–75%) [4] and specificity (40–82%) [5], routine transrectal ultrasound (TRUS)-guided systematic biopsy (SB) remains the diagnostic standard for PCa [6].

The ability to precisely detect PCa using magnetic resonance imaging (MRI) has led to the development of software-assisted MRI–ultrasound fusion guided targeted biopsy (TB). The European Association of Urology (EAU) guidelines currently recommend the combination of TB and SB as the first-line biopsy method for patients with suspected PCa with an abnormal MRI [7]. SB + TB indeed captures an additional number of PCa, but it does so at the expense of a concomitant increase in biopsy cores and overdetection of clinically insignificant prostate cancer (ciPCa) [8, 9]. The more the biopsy cores, the higher the complication rates, such as hematuria and urinary retention [10]. Besides, overdiagnosis and the following unnecessary treatment of low-grade PCa bear heavily on patients [9]. Thus, exploring a new biopsy method that could achieve an acceptable clinically significant prostate cancer (csPCa) detection rate with fewer cores is important.

A previous study has demonstrated that ipsilateral SB (ipsi-SB) + TB could detect more patients with csPCa than contralateral SB (contra-SB) + TB [11]. However, the study was performed on a single definition of csPCa. At present, no universally accepted definition of csPCa exists [12]. Therefore, we performed this study to evaluate the csPCa detection rate of various prostate sampling schemes and verify whether ipsi-SB + TB or contra-SB + TB could achieve almost equal csPCa detection rates to SB + TB using fewer cores based on different csPCa definitions.

## Methods

### Patient selection

**Fig. 1:**
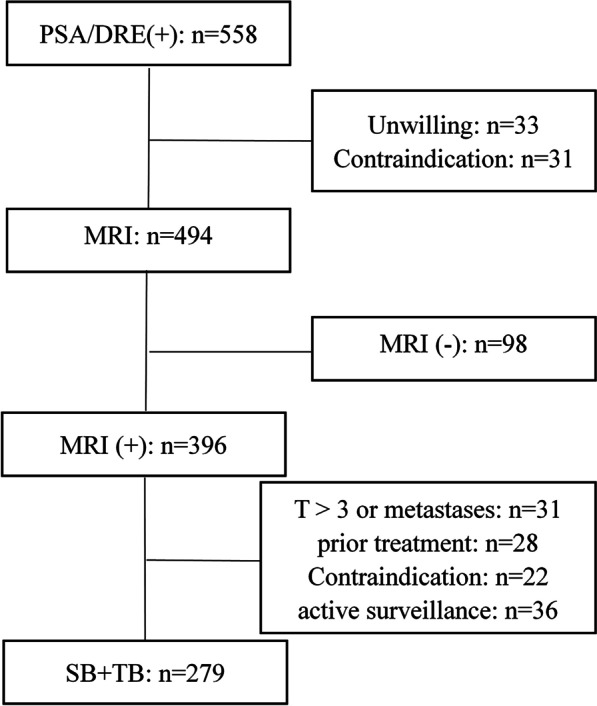
Flowchart for study inclusion/exclusion. PSA = prostate-specific antigen; DRE = digital rectal examination; MRI = magnetic resonance imaging; SB = systematic biopsy; TB = targeted biopsy

Men with increased serum prostate-specific antigen (PSA) levels (PSA > 4 ng/mL) or an abnormal digital rectal examination (DRE) underwent 3.0-T prostate MRI. We included patients whose MRI was positive [with at least one lesion with a Prostate Imaging Reporting and Data System (PI-RADS) score of 3 or greater]. Patients with clinical stage T > 3 or metastases, prior treatment for PCa, and under active surveillance were excluded from the study (Fig. 1).

### Multiparametric MRI

Multiparametric MRI was performed using a 3.0-T scanner with a 32-channel surface coil (Ingenia, Philips, Netherlands). In a nutshell, the study involved triplanar T2-weighted imaging, diffusion-weighted imaging with a b value of 0–100–1000–2000 s/mm^2^, apparent diffusion coefficient maps (calculated by the b value of 100–1000 s/mm^2^ automatically), and dynamic contrast (gadolinium, 2.5 mL/s, 0.1 mmol/kg)-enhanced imaging sequences according to the minimum standards set by consensus guidelines [10]. One genitourinary radiologist interpreted all the lesions visible in MRI according to the PI-RADS version 2 on a scale from 1 (no suspicion) to 5 (high suspicion).

### Biopsy procedure

A fluoroquinolone antibiotic was prescribed 3 days before biopsy to prevent postoperative infection, and an enema was generally performed. A MyLab Twice ultrasound system was used with an EC-123 7.5-MHz transrectal end-fire probe (EsaoteSpA, Genova, Italy) accompanied by an automatic biopsy gun with an 18-G needle for sampling.

### TB procedure

MRI-TRUS registration (i.e., matching of the previously obtained suspicious MRI lesions with the real-time image of the prostate during TRUS biopsy) was performed by software-assisted rigid registration Virtual Navigator (Esaote, Genoa, Italy). Each MRI suspicious lesion was biopsied with at least two cores.

### SB procedure

**Fig. 2:**
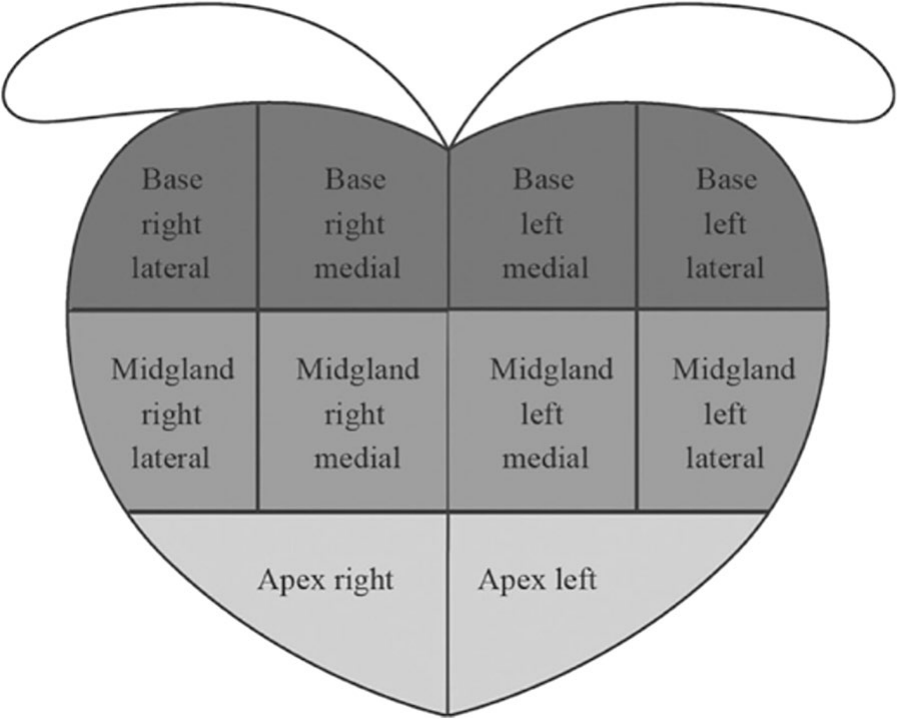
The standard 10-core biopsy. 10 cores obtained from the lateral and medial aspects of the base and midgland and apical prostate of the left and right side. (Reprinted with the kind permission from Ma et al., 2017 [13])

The MRI overlay TB was then removed, and a second physician performed an SB with ultrasonographic guidance alone. The standard 10-core biopsy was obtained from the lateral and medial aspects of the base and midgland, and the apical prostate of the left and right sides [13] (Fig. 2).

### Hypothetical biopsy sampling schemes and different definitions of csPCa

We hypothesized four biopsy sampling schemes in this study: SB only, TB only, ipsi-SB + TB, and contra-SB + TB. Among these, SB + TB was regarded as the reference. csPCa was defined according to the EAU guidelines [International Society for Urological Pathology (ISUP) 2 or higher, definition 1 (D1)] [14] or the Epstein criteria [Gleason score (GS) > 6 or GS 6 with ≥ 50% of cancer per core involvement or > 2 cores with cancer, definition 2 (D2)] [15].

### Outcomes of interest

A comparison of the csPCa detection rate of the four hypothetical biopsy sampling schemes based on different csPCa definitions was our primary endpoint. The secondary endpoint was to assess the diagnostic concordance and upgrading between the aforementioned sampling schemes and SB + TB.

### Statistical analysis

Data were prospectively collected according to the Standards of Reporting for MRI-targeted Biopsy Studies database [16]. Descriptive statistics were used to describe the patient characteristics. The difference in the needed cores of different biopsy methods was compared by employing the Wilcoxon signed-rank test. We compared the csPCa detection rate of different biopsy strategies head-to-head using the McNemar test. A Cochran’s Q test was used for comparing the pathological concordance and upgrading between different biopsy methods. We evaluated the potential predictors of biopsy result upgrading using multivariable logistic regression. All statistical analyses were conducted using SPSS, version 22.0, and a statistical significance level of 5% was used.

## Results

### Baseline characteristics of patients

In this prospective single-center diagnostic study, 279 subjects with a median age of 71 years [interquartile range (IQR): 62–80] and median PSA of 10.04 ng/mL (IQR: 6.38–18.00) were enrolled from January 2017 to December 2020 irrespective of the biopsy history. Abnormal DRE was found in 74 men (26.52%). The patients’ demographics are given in Table 1.

**Table 1:** Patients’ baseline characteristics, TRUS findings and MRI findings

Men, no	279
Age, year (IQR)	71 (65–77)
PSA, ng/mL (IQR)	10.04 (6.38–18.00)
Suspicious DRE findings, n (%)	74 (26.52)
TRUS prostate volume, mL (IQR)	57.00 (41.00–82.30)
Men with prior biopsy, n (%)	89 (31.90)
Men without biopsy history, n(%)	190 (68.10)
Abnormal TRUS findings, n (%)	139 (49.82)
Urologists’ biopsy experience, year (IQR)	4 (4–5)
MRI suspicious lesions per patient, no. (IQR)	1 (1–1)
Total lesions, no	353
PIRADS v2 score, n (%)	
3	113 (32.01)
4	169 (47.88)
5	71 (20.11)
Location	
Peripheral zone, n (%)	232 (65.72)
Transitional zone, n (%)	121 (34.28)

Values are presented as median (interquartile range [IQR]). Statistically significant at *P* < 0.05. TRUS, transrectal ultrasound; MRI, magnetic resonance imaging; PSA, prostate-specific antigen; DRE, digital rectal examination; PIRADS, prostate imaging reporting and data system

### Biopsy cores

The median core of SB, TB, ipsi-SB + TB, and contra-SB + TB was 10, 2, 7, and 7, respectively; they all differed significantly from SB + TB (12, P < 0.001) (Table 2). Obviously, TB showed the best detection of csPCa for the total number of cores regardless of the definition (P < 0.001) and SB performed the worst (P < 0.001). A comparison of the csPCa positive core rates of ipsi-SB + TB and contra-SB + TB revealed that the former performed better irrespective of the definition of csPCa (P < 0.001) (Table 2).

**Table 2:** Summary of biopsy cores

	SB	TB	ipsi-SB + TB	contra-SB + TB	SB + TB
Biopsy cores, no	10 (10–10)	2 (2–2)	7 (7–7)	7 (7–7)	12 (12–12)
Positive biopsy cores, no	1 (0–4)	0 (0–2)	2 (0–5)	2 (0–3)	2 (0–6)
D1 positive core rate, n (%)	373 (10.69)	230 (29.72)	509 (20.21)	324 (12.86)	603 (14.14)
P	< 0.001	< 0.001	< 0.001	0.017	–
D2 positive core rate, n (%)	678 (19.43)	357 (46.12)	817 (32.43)	575 (22.83)	1035 (24.27)
P	< 0.001	< 0.001	< 0.001	0.051	–

Values are presented as median (interquartile range). Statistically significant at P < 0.05. SB, systematic biopsy; TB, targeted biopsy; ipsi-SB, ipsilateral SB; contra-SB, contralateral SB; D1, definition 1 (EAU guidelines); csPCa, clinically significant prostate cancer; D2, definition 2 (Epstein criteria)

### PCa detection rates

On the basis of D1, 104 and 90 patients with csPCa were detected by ipsi-SB + TB and contra-SB + TB, respectively (P = 0.042). And on the basis of D2, both ipsi-SB + TB and contra-SB + TB detected 146 patients with csPCa (P = 1.000). SB could detect more patients with csPCa than TB when used alone; however, the difference was insignificant on the grounds of D1 (D1: 82 vs. 80, P = 0.302; D2: 143 vs. 118, P = 0.002). Compared with SB + TB, each remaining method detected significantly fewer patients with csPCa regardless of the definition of csPCa (P < 0.001) except ipsi-SB + TB, which achieved almost the same csPCa detection rate as that of SB + TB based on D1 (P = 0.066) (Table 3). SB, TB, ipsi-SB + TB, and contra-SB + TB detected 61, 38, 42, and 56 patients with PCa, respectively, which were clinically insignificant when csPCa was defined as D1. It was obvious that TB (P = 0.018) and ipsi-SB + TB (P = 0.021) detected significantly fewer patients with ciPCa compared with SB + TB (55).

**Table 3:** Detection rates of csPCa

	SB	TB	ipsi-SB + TB	contra-SB + TB	SB + TB
Detected D1 csPCa cases, n (%)	82 (29.39)	80 (28.67)	104 (37.28)	90 (32.26)	106 (37.99)
P	< 0.001	< 0.001	0.066	< 0.001	-
Detected D2 csPCa cases, n (%)	143 (51.25)	118 (42.29)	146 (52.33)	146 (52.33)	161 (57.71)
P	< 0.001	< 0.001	< 0.001	< 0.001	-

Statistically significant at *P* < 0.05. csPCa, clinically significant prostate cancer; SB, systematic biopsy; TB, targeted biopsy; ipsi-SB, ipsilateral SB; contra-SB, contralateral SB; D1, definition 1 (EAU guidelines); D2, definition 2 (Epstein criteria); GS, Gleason score

### GS distribution, concordance, and upgrading

**Fig. 3:**
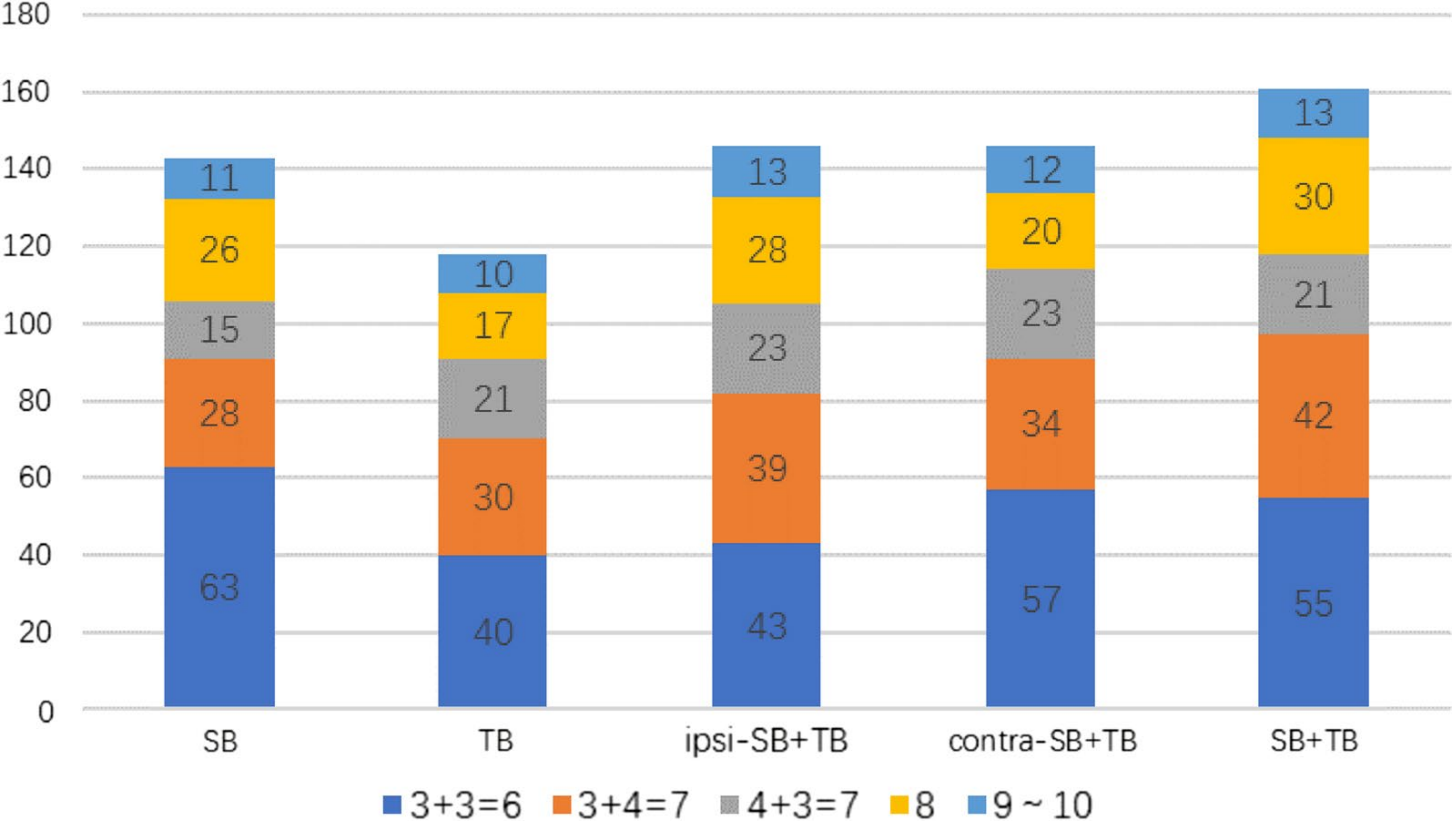
Distribution of the Gleason score on each biopsy method

**Fig. 4:**
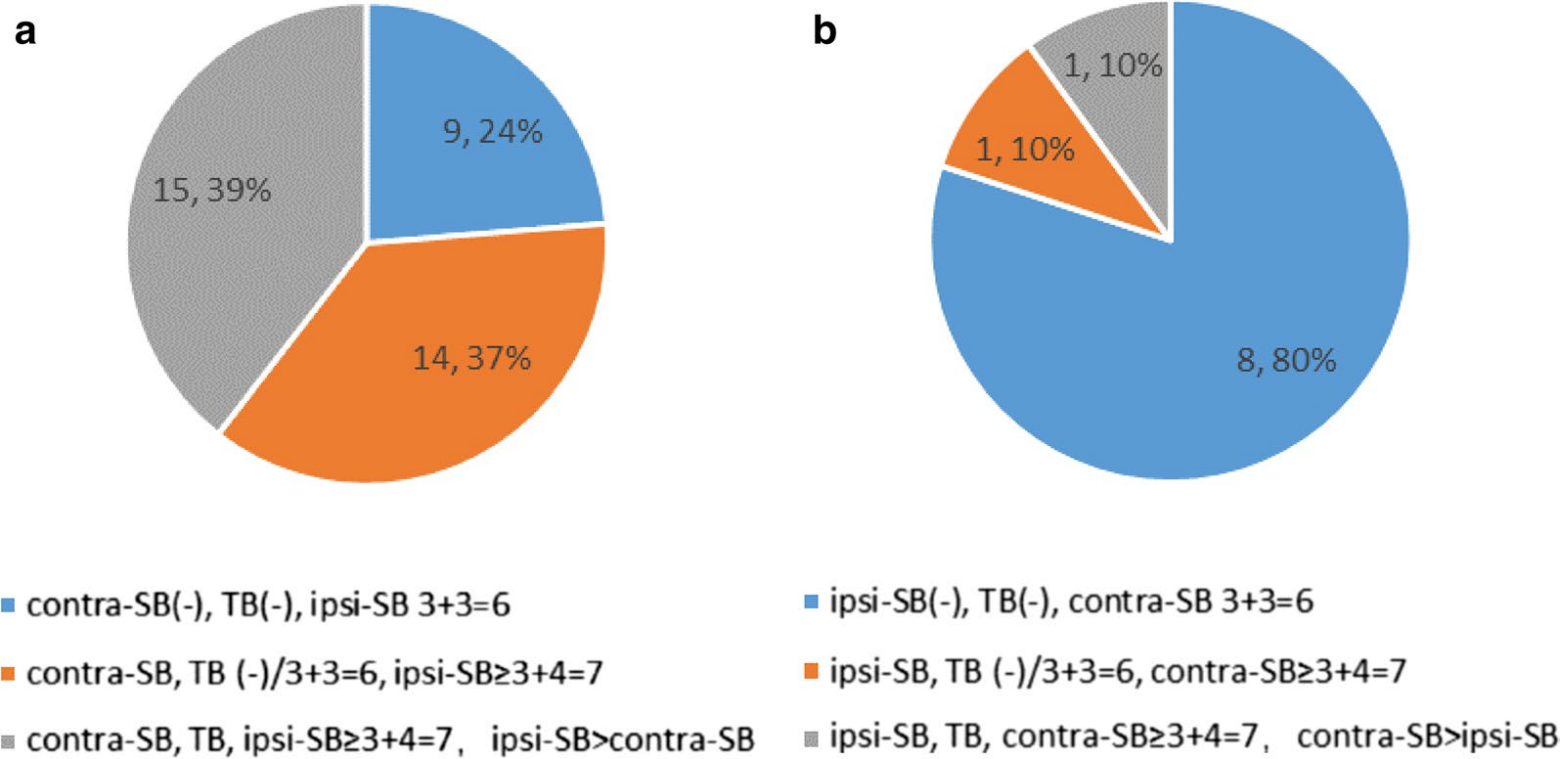
Gleason score concordance and upgrading seen (**a**) on ipsi-SB and (**b**) on contra-SB

The distribution of the GS on each biopsy method could be seen in Fig. 3. It is worth noting that the number of PCa with a GS of 6 detected by SB was more than that by TB (P < 0.001), but the number of PCa with a GS of ≥ 7 detected by both of them was almost equal (P = 0.311). ipsi-SB + TB identified the same number of PCa as that of contra-SB + TB (P = 1.000) but higher number of patients with a GS of ≥ 7 (P < 0.001) and fewer patients with a GS of 6 (P < 0.001).

ipsi-SB detected 92 patients with a higher GS, and 38 patients were still detected after combining with TB. Of the 38 patients, 9 had a GS of 6, 14 had a GS of ≤ 6 on contra-SB + TB to ≥ 3 + 4 on ipsi-SB + TB, and the remaining 15 were concordant patients (Fig. 4a). The upgrading of a patient from a GS of ≤ 6 in one biopsy method to higher than a GS of ≤ 6 in another was considered as insignificantly upgraded. A patient upgrading from a GS of ≤ 6 in one biopsy method to a GS of ≥ 3 + 4 in another was considered as significantly upgraded. A patient upgrading from a GS of ≥ 3 + 4 in one biopsy method to higher than a GS of ≥ 3 + 4 in another was considered concordant. Details of the 38 upgrading patients on ipsi-SB are summarized in Additional file 1: Table S1.

In contrast, 17 subjects were identified with a higher GS on contra-SB compared with ipsi-SB, and only 10 additional upgrades occurred after combining with TB. Among them, eight patients had a GS of 6, one had a GS of 6 on ipsi-SB + TB to a GS of 8 on contra-SB + TB, and one had a GS of 9 on ipsi-SB + TB to a GS of 10 on contra-SB + TB (Fig. 4b). Details of the 10 upgrading patients on contra-SB are summarized in Additional file 2: Table S2.

### Potential predictors of GS upgrading on ipsi-SB + TB and contra-SB + TB

We evaluated the potential predictors of GS upgrading on ipsi-SB + TB and contra-SB + TB. For all 38 patients with a higher GS on ipsi-SB + TB, decreased TRUS prostate volume, prior biopsy history, lesion located in the peripheral zone (PZ), and higher PSA level were associated with GS upgrading. Among them, prior biopsy history had the strongest association with GS upgrading [odds ratio (OR): 2.365; P = 0.008] (Table 4). In the 14 significantly upgrading patients on ipsi-SB + TB, decreased TRUS prostate volume and lesion located in the PZ remained significant (Table 4).

**Table.4:** Associated predictors of Gleason score upgrading on ipsi-SB + TB

		38 upgrading				14 significantly upgrading	
	OR	95% CI	*P* value		OR	95% CI	*P* value
TRUS prostate volume (per 10 volume)	0.970	0.950–0.990	0.004		0.980	0.950–1.012	0.022
Biopsy history (yes or no)	2.365	0.903–6.192	0.008		–	–	–
Location (PZ or TZ)	1.949	0.713–5.324	0.019		8.424	1.201–59.065	0.032
PSA (per ng/mL)	1.001	0.999–1.004	0.028		–	–	–

Statistically significant at *P* < 0.05. SB, systematic biopsy; TB, targeted biopsy; ipsi-SB, ipsilateral SB; OR, odds ratio; CI, confidence interval; TRUS, transrectal ultrasound; PZ, peripheral zone; TZ, transitional zone; PSA, prostate-specific antigen

Prior biopsy history (OR: 3.148; P = 0.021) and inadequate biopsy experience (OR: 0.701; P = 0.032) were associated with a GS of 10 upgrading on contra-SB + TB (Table 5).The basic characteristics of the only patient with significant upgrading on contra-SB + TB were as follows: age 82 years, PSA 30.24 ng/mL, DRE ( +), TRUS prostate volume 66.9 mL, prior biopsy history, abnormal TRUS findings, urologists’ biopsy experience 5, MRI suspicious lesions 1, maximum PI-RADS 5, and lesion position PZ.

**Table.5:** Associated predictors of 10 Gleason score upgrading on contra-SB + TB

	OR	95% CI	*P* value
Biopsy history (yes or no)	3.148	0.527–18.802	0.021
Urologists’ biopsy experience (per year)	0.701	0.349–1.409	0.032

Statistically significant at *P* < 0.05. SB, systematic biopsy; TB, targeted biopsy; contra-SB, contralateral SB; OR, odds ratio; CI, confidence interval

## Discussion

SB is a relatively cost-effective and nonoperator-dependent method of detecting PCa and does not need specialized equipment. However, the method suffers from relatively lower diagnostic accuracy and more biopsy cores. Despite several limitations, systematic sampling of the prostate with different core numbers (commonly 10–12 cores) still represents an integral aspect of diagnosing or excluding PCa [17]. The MRI pathway (MRI with or without TB) has been increasingly used for the detection and risk stratification of csPCa [18, 19]. Van der Leest M et al. [20] concluded that MRI-TB had an identical detection rate of csPCa and significantly fewer ciPCa using fewer needles compared with SB.

In this study, TB indeed had a significantly higher csPCa positive core rate than SB; however, SB still could detect significantly more patients with csPCa than TB at least based on D2. Similar results were reported by Hakozaki et al. [21] with a higher csPCa detection rate in the SB group; they defined csPCa according to D2 in this context. More patients with csPCa were also detected by SB than by TB on the grounds of D1, although the difference was insignificant in this article. This finding was compatible with that of a previous similar study (Radtke et al. [22]), which reported that the csPCa (in line with D1 in our study) detection rate was higher using SB alone than using TB alone. Compared with D1, D2 of csPCa is more inclusive. For example, cancer with a GS of 6 and three cores would be considered ciPCa on the grounds of D1 but csPCa based on D2. In the series by Filson et al. [23], TB offered the potential to identify more higher-risk patients with PCa. As a consequence, although SB still detected more patients with csPCa than TB, the difference was insignificant when using the stricter D1 of csPCa. Therefore, TB was nonsuperior to SB in terms of detecting csPCa at least in the current study.

Recently, many different modified sampling schemes have emerged to increase the detection of aggressive tumors and decrease biopsy cores and concomitant complications [24]. A regional TB strategy (10.58 cores) proposed by Raman et al. [25] detected a similar number of patients with csPCa to SB + TB. And many other studies [26, 27] also focused on optimizing the number of cores sampled from the targeted area and reached the conclusion that saturation TB (10–20 cores) detected as many patients with csPCa as 20- to 26-core SB + TB. However, omitting SB or not was still in dispute, and the scheme of saturation TB was not unified. In an early study, Ploussard et al. [28] evaluated the added value of concomitant SB for predicting the final grade group in patients with positive MRI findings who underwent TB. The results showed that SB reclassified a non-negligible proportion of patients in a higher-risk category and modified the final treatment decision-making. As a consequence, SB should not be omitted at least in MRI-positive cases, just identical to the EAU guidelines [7]. Although SB + TB indeed led to the detection of more PCa and csPCa among patients with MRI-visible lesions, the cores were also increased [8].

Bryk et al. [9] enrolled a cohort of 211 men with a single unilateral suspicious lesion on MRI and recommended ipsi-SB + TB, as the detection of csPCa increased with only a modest increase in ciPCa detection. Our findings were consistent with those of Bryk et al. when we used nearly the same definition of csPCa (D1). ipsi-SB + TB also performed better in terms of a positive core rate than contra-SB + TB irrespective of the definition of csPCa. However, the two methods had an equivalent detection of csPCa based on D2 in this study. Thus, no matter which csPCa definition was chosen in this study, ipsi-SB + TB did not perform worse than contra-SB + TB in the detection of patients with csPCa.

In this study, both ipsi-SB + TB and contra-SB + TB detected fewer patients with csPCa regardless of the definition of csPCa when compared with SB + TB. The aforementioned findings were identical with those of Freifeld et al. [11], who found that patients were more or less missed or misclassified by TB alone, TB + ipsi-SB, and TB + contra-SB. However, slightly different from the previous study, ipsi-SB + TB detected an almost equal number of patients with csPCa as SB + TB based on D1 in this study. This provided us a novel biopsy scheme that could acquire an equivalent csPCa detection value to SB + TB using significantly fewer cores.

Further, we analyzed the specific GS of each biopsy method. Also, we found that ipsi-SB + TB identified the same number of PCa as contra-SB + TB did but with a higher number of patients with a GS of ≥ 7 and fewer patients with a GS of 6. With respect to the upgrading condition, combining ipsi-SB and contra-SB + TB led to 38 GS upgrading; however, combining contra-SB and ipsi-SB + TB only resulted in 10 GS upgrading. Also, a large number of the 38 patients upgraded from GS ≤ 6 to GS ≥ 7; conversely, 80% of the 10 cases with no cancer on ipsi-SB + TB were diagnosed with PCa with a GS of 6 when combined with contra-SB. Recently, as a result of the widespread use of PSA testing, the incidence of PCa has increased (including ciPCa) [29]. Also, after many years of aggressive treatment of PCa, the reduced overdiagnosis and overtreatment of ciPCa have caught the attention of the urology community [30]. According to the EAU guidelines [14], active surveillance should be discussed for patients at low risk of PCa (PSA < 10 ng/mL and GS < 7 and cT1-2a). Patients at intermediate risk (PSA 10–20 ng/mL or GS 7 or cT2b) and those at high risk of PCa (PSA > 20 ng/mL or GS > 7 or cT2c) are strongly recommended to undergo radical prostatectomy. As a result, additional contra-SB results in the overdetection of low-risk PCa, while additional ipsi-SB is more likely to change the patients’ recommended treatment strategy.

This study has some limitations. First, the inclusion criteria of this study are confined to patients with abnormal MRI; thus, no statement of cancer missed in the initial MRI could be made. Siddiqui et al. [31] reported that MRI showed a negative predictive value of 98% for PCa with a GS of 7 or greater; hence, further studies are needed to explore the negative predictive value of MRI. Second, whole-mount histopathology was not the reference specimen when the cancer detection rate was compared. Third, the rigid registration system did not allow us to make adjustments; even deformations happened to the prostate by the TRUS probe, although some anatomical landmarks can be used to make cognitive fusion at that time in our study [32]. Finally, the same patient was tested with SB and TB, and biopsy complications, such as hemorrhage and swelling of the first conducted one-TB procedure might have negatively affected the SB.

## Conclusions

Ipsi-SB + TB could acquire an almost equivalent csPCa detection value to SB + TB using significantly fewer cores when csPCa was defined according to the EAU guidelines. Given that there was only one significantly upgrading patient on contra-SB, our results suggested that contra-SB could be avoided.

## Supplementary Information

**Additional file 1. Table S1** Detail of the 38 upgrading patients on ipsi-SB.

**Additional file 2. Table S2** Detail of the 10 upgrading patients on contra-SB.

## Data Availability

All data generated or analyzed during this study are included in this published article and its supplementary information files.

## Abbreviations

PCa: Prostate cancer
TRUS: Transrectal ultrasound
SB: Systematic biopsy
MRI: Magnetic resonance imaging
TB: Targeted biopsy
EAU: European Association of Urology
ciPCa: Clinically insignificant prostate cancer
csPCa: Clinically significant prostate cancer
ipsi-SB: Ipsilateral SB
contra-SB: Contralateral SB
PSA: Prostate-specific antigen
DRE: Digital rectal examination
PI-RADS: Prostate Imaging Reporting and Data System
ISUP: International Society for Urological Pathology
GS: Gleason score
IQR: Interquartile range
PZ: Peripheral zone
TZ: Transitional zone
OR: Odds ratio
