# Supplementary material for: Clinically significant prostate cancer (csPCa) detection with various prostate sampling schemes based on different csPCa definitions
Source: BMC Urol. 2021 Dec 23;21:183. doi: 10.1186/s12894-021-00949-7 (PMC8697444; doi:10.1186/s12894-021-00949-7)
Supplement: Supplementary file 1 — Additional file 1. Table S1 Detail of the 38 upgrading patients on ipsi-SB. [file 12894_2021_949_MOESM1_ESM.docx]

**Additional file 1: Table 1** Detail of the 38 upgrading patients on ipsi-SB.

| **Patient id** | **ipsi-SB GS** | **contra-SB GS** | **TB GS** | **SB+TB GS** |
| --- | --- | --- | --- | --- |
| **1** | 4+4=8 | - | 4+3=7 | 4+4=8 |
| **2** | 4+3=7 | - | 3+4=7 | 4+3=7 |
| **3** | 5+4=9 | 3+3=6 | 4+5=9 | 5+4=9 |
| **4** | 4+3=7 | 3+4=7 | 3+4=7 | 4+3=7 |
| **5** | 4+4=8 | 3+3=6 | 4+3=7 | 4+4=8 |
| **6** | 4+4=8 | - | 3+4=7 | 4+4=8 |
| **7** | 4+4=8 | 3+3=6 | 4+3=7 | 4+4=8 |
| **8** | 4+4=8 | 3+4=7 | 4+3=7 | 4+4=8 |
| **9** | 5+4=9 | - | 4+5=9 | 5+4=9 |
| **10** | 3+4=7 | 3+3=6 | - | 3+4=7 |
| **11** | 4+4=8 | - | 4+3=7 | 4+4=8 |
| **12** | 4+4=8 | 3+4=7 | 4+3=7 | 4+4=8 |
| **13** | 4+4=8 | 3+3=6 | 4+3=7 | 4+4=8 |
| **14** | 5+4=9 | - | 4+5=9 | 5+4=9 |
| **15** | 5+5=1- | - | 4+5=9 | 5+5=1- |
| **16** | 4+3=7 | 4+3=7 | 3+3=6 | 4+3=7 |
| **17** | 3+4=7 | 3+3=6 | 3+3=6 | 3+4=7 |
| **18** | 3+4=7 | 3+3=6 | - | 3+4=7 |
| **19** | 3+4=7 | - | 3+3=6 | 3+4=7 |
| **20** | 3+4=7 | 3+3=6 | 3+3=6 | 3+4=7 |
| **21** | 4+3=7 | - | 3+3=6 | 4+3=7 |
| **22** | 3+4=7 | - | 3+3=6 | 3+4=7 |
| **23** | 4+3=7 | - | 3+3=6 | 4+3=7 |
| **24** | 4+5=9 | 3+3=6 | - | 4+5=9 |
| **25** | 4+3=7 | - | - | 4+3=7 |
| **26** | 3+4=7 | 3+3=6 | 3+3=6 | 3+4=7 |
| **27** | 4+4=8 | - | 3+3=6 | 4+4=8 |
| **28** | 3+4=7 | 3+3=6 | 3+3=6 | 3+4=7 |
| **29** | 4+4=8 | - | - | 4+4=8 |
| **30** | 3+3=6 | - | - | 3+3=6 |
| **31** | 3+3=6 | - | - | 3+3=6 |
| **32** | 3+3=6 | - | - | 3+3=6 |
| **33** | 3+3=6 | - | - | 3+3=6 |
| **34** | 3+3=6 | - | - | 3+3=6 |
| **35** | 3+3=6 | - | - | 3+3=6 |
| **36** | 3+3=6 | - | - | 3+3=6 |
| **37** | 3+3=6 | - | - | 3+3=6 |
| **38** | 3+3=6 | - | - | 3+3=6 |

ipsi-SB=ipsilateral SB; SB=systematic biopsy; GS= Gleason score; contra-SB=contralateral SB; TB=targeted biopsy.
