# Supplementary material for: Clinically significant prostate cancer (csPCa) detection with various prostate sampling schemes based on different csPCa definitions
Source: BMC Urol. 2021 Dec 23;21:183. doi: 10.1186/s12894-021-00949-7 (PMC8697444; doi:10.1186/s12894-021-00949-7)
Supplement: Supplementary file 2 — Additional file 2. Table S2 Detail of the 10 upgrading patients on contra-SB. [file 12894_2021_949_MOESM2_ESM.docx]

**Additional file 2: Table 2** Detail of the 10 upgrading patients on contra-SB.

| **Patient id** | **ipsi-SB GS** | **contra-SB GS** | **TB GS** | **SB+TB GS** |
| --- | --- | --- | --- | --- |
| **1** | - | 3+3=6 | - | 3+3=6 |
| **2** | - | 3+3=6 | - | 3+3=6 |
| **3** | - | 3+3=6 | - | 3+3=6 |
| **4** | - | 3+3=6 | - | 3+3=6 |
| **5** | - | 3+3=6 | - | 3+3=6 |
| **6** | - | 3+3=6 | - | 3+3=6 |
| **7** | - | 3+3=6 | - | 3+3=6 |
| **8** | - | 3+3=6 | - | 3+3=6 |
| **9** | 4+5=9 | 5+5=10 | 4+5=9 | 5+5=10 |
| **10** | 3+3=6 | 4+4=8 | 0 | 4+4=8 |

contra-SB=contralateral SB; SB=systematic biopsy; ipsi-SB=ipsilateral SB; GS= Gleason score; TB=targeted biopsy.
